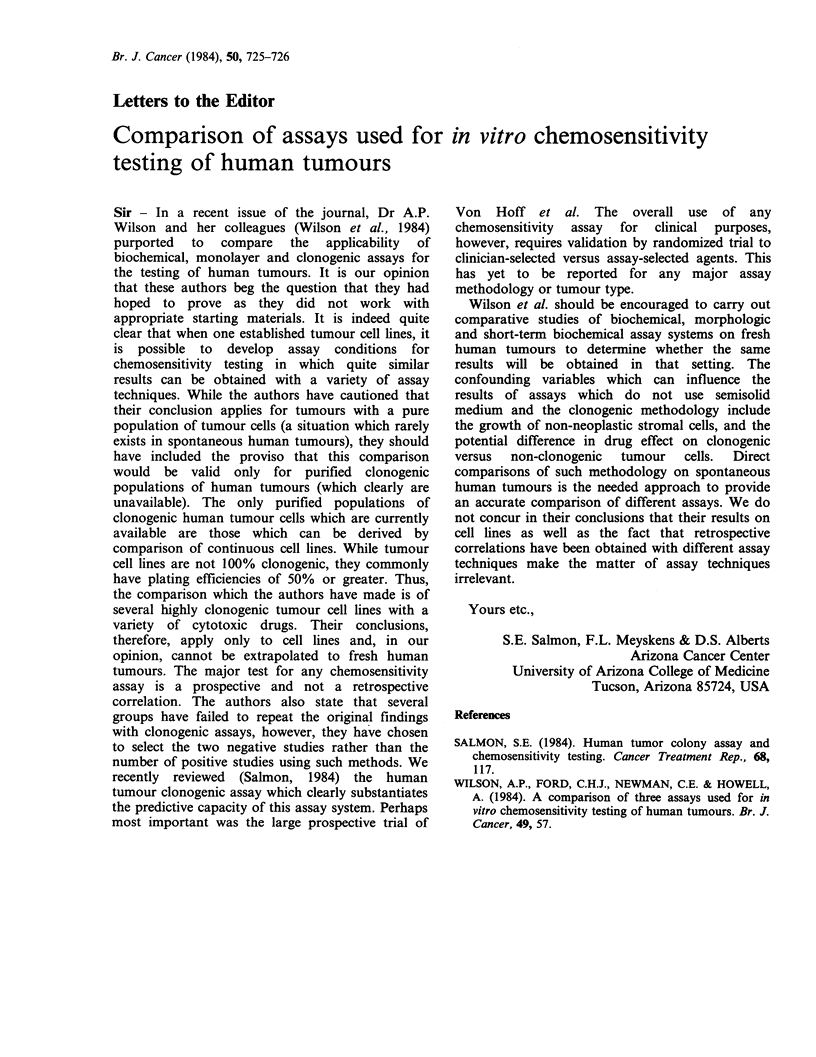# Comparison of assays used for in vitro chemosensitivity testing of human tumours.

**DOI:** 10.1038/bjc.1984.243

**Published:** 1984-11

**Authors:** S. E. Salmon, F. L. Meyskens, D. S. Alberts


*Br. J. Cancer* (1984), **50**, 725–726

### Letters to the Editor

### Comparison of assays used for *in vitro* chemosensitivity testing of human tumours

**Sir** – In a recent issue of the journal, Dr A.P. Wilson and her colleagues (Wilson *et al.,* 1984) purported to compare the applicability of biochemical, monolayer and clonogenic assays for the testing of human tumours. It is our opinion that these authors beg the question that they had hoped to prove as they did not work with appropriate starting materials. It is indeed quite clear that when one established tumour cell lines, it is possible to develop assay conditions for chemosensitivity testing in which quite similar results can be obtained with a variety of assay techniques. While the authors have cautioned that their conclusion applies for tumours with a pure population of tumour cells (a situation which rarely exists in spontaneous human tumours), they should have included the proviso that this comparison would be valid only for purified clonogenic populations of human tumours (which clearly are unavailable). The only purified populations of clonogenic human tumour cells which are currently available are those which can be derived by comparison of continuous cell lines. While tumour cell lines are not 100% clonogenic, they commonly have plating efficiencies of 50% or greater. Thus, the comparison which the authors have made is of several highly clonogenic tumour cell lines with a variety of cytotoxic drugs. Their conclusions, therefore, apply only to cell lines and, in our opinion, cannot be extrapolated to fresh human tumours. The major test for any chemosensitivity assay is a prospective and not a retrospective correlation. The authors also state that several groups have failed to repeat the original findings with clonogenic assays, however, they have chosen to select the two negative studies rather than the number of positive studies using such methods. We recently reviewed (Salmon, 1984) the human tumour clonogenic assay which clearly substantiates the predictive capacity of this assay system. Perhaps most important was the large prospective trial of Von Hoff *et al.* The overall use of any chemosensitivity assay for clinical purposes, however, requires validation by randomized trial to clinician-selected versus assay-selected agents. This has yet to be reported for any major assay methodology or tumour type.

Wilson *et al.* should be encouraged to carry out comparative studies of biochemical, morphologic and short-term biochemical assay systems on fresh human tumours to determine whether the same results will be obtained in that setting. The confounding variables which can influence the results of assays which do not use semisolid medium and the clonogenic methodology include the growth of non-neoplastic stromal cells, and the potential difference in drug effect on clonogenic versus non-clonogenic tumour cells. Direct comparisons of such methodology on spontaneous human tumours is the needed approach to provide an accurate comparison of different assays. We do not concur in their conclusions that their results on cell lines as well as the fact that retrospective correlations have been obtained with different assay techniques make the matter of assay techniques irrelevant.

Yours etc.,

S.E. Salmon, F.L. Meyskens & D.S. Alberts
Arizona Cancer Center
University of Arizona College of Medicine
Tucson, Arizona 85724, USA

### References

SALMON, S.E. (1984). Human tumor colony assay and chemosensitivity testing. *Cancer Treatment Rep.*, **68**, 117.

WILSON, A.P., FORD, C.H.J., NEWMAN, C.E. & HOWELL, A. (1984). A comparison of three assays used for *in vitro* chemosensitivity testing of human tumours. *Br. J. Cancer,* **49**, 57.